# The effect of an electronic medical record intervention on hydroxychloroquine prescribing habits and surveyed providers’ opinions of the 2016 American Academy of Ophthalmology guidelines in the rheumatology and dermatology practices of an academic institutionle

**DOI:** 10.1186/s12913-021-06954-8

**Published:** 2021-09-03

**Authors:** Rebecca S. Overbury, Gregory J. Stoddard, Jakrapun Pupaibool, Christopher B. Hansen, Dorota Lebiedz-Odrobina

**Affiliations:** 1 Department of Internal Medicine, Division of Rheumatology, University of Utah, School of Medicine, 30 North 1900 East, 4B200, Salt Lake City, UT 84132 USA; 2 Department of Internal Medicine, University of Utah, Salt Lake City, USA; 3 Department of Internal Medicine, Division of Infectious Diseases, University of Utah, Salt Lake City, USA; 4 Department of Dermatology, University of Utah, Salt Lake City, USA

**Keywords:** Hydroxychloroquine, Guidelines, Disease-modifying anti-rheumatic drugs, Drug toxicity

## Abstract

**Background:**

Retinal toxicity is a rare adverse event related to the use of hydroxychloroquine (HCQ). To address this, in 2016, the American Academy of Ophthalmology (AAO) issued guidelines recommending that HCQ not exceed 5 mg/kg/day. We analyzed HCQ prescribing habits at our institution, compared to these guidelines, and used surveys to determine the opinions on these guidelines. We then introduced, in a prospective and non-controlled study, a clinical decision support (CDS) tool into the electronic medical record (EMR) to study how this intervention might affect adherence with or opinions on these guidelines.

**Methods:**

Data were collected pre-intervention (June 2017–January 2019) and post-intervention (March 2019–April 2020). In January 2019 we released our CDS tool. Results were analyzed using descriptive statistics for demographic data and Fisher’s exact tests for comparisons of proportions between groups.

**Results:**

Pre-intervention, we reviewed 1128 rheumatology charts and 282 dermatology charts. 31.0 and 39.7% respectively (32.8% combined) were prescribed HCQ > 5 .0 mg/kg/day. Post-intervention, we reviewed 1161 rheumatology charts and 110 dermatology charts. 23.0 and 25.5% respectively (23.2% combined) were prescribed HCQ > 5.0 mg/kg/day. Post-intervention, 9.6% fewer patients were prescribed HCQ > 5 mg/kg/day (*P* < .001). Pre-intervention, we compiled 18 rheumatology surveys and 12 dermatology surveys. Post-intervention, we compiled 16 rheumatology surveys and 12 dermatology surveys. Post-intervention, fewer rheumatologists incorrectly described the AAO weight-based guidelines. Combined, there was an overall reduction but not of statistical significance (*P* = .47). The majority of providers surveyed believed that the CDS tool was useful (72.2%).

**Conclusions:**

At our academic institution, there remains unfamiliarity with and hesitation to comply with the 2016 AAO guidelines. Prescribed doses often exceed what is recommended in these guidelines. A CDS tool can improve adherence with these guidelines and might improve providers’ familiarity with these guidelines.

**Supplementary Information:**

The online version contains supplementary material available at 10.1186/s12913-021-06954-8.

## Background

Hydroxychloroquine (HCQ) is a commonly used medication in the fields of rheumatology and dermatology. This medication is an immunomodulator and is considered a disease modifying anti-rheumatic drug (DMARD). It is used to treat rheumatoid arthritis (RA), systemic lupus erythematosus (SLE), and a variety of other autoimmune skin and systemic inflammatory conditions. HCQ is hypothesized to have a therapeutic benefit in autoimmune disease through several mechanisms. First it has an inhibitory effect on several toll-like receptors (TLRs), which disrupts co-stimulation of B-cells and antigen processing [1–5]. It also exerts a lysosomotropic effect on sub-cellular compartments affecting the acidity of endosomal compartments and thereby more broadly inhibiting normal pH-dependent processing of proteins and ligands crucial to the immune response [6–9].

HCQ is generally well tolerated by patients and has a good safety profile [10]. However, a rare but devastating adverse event related to the use of HCQ is ophthalmologic toxicity in the form of retinopathy. If the HCQ retinopathy goes undetected, it can progress to cause vision deficits and blindness [11, 12]. This retinal toxicity is irreversible, however if diagnosed early, there is only mild and limited progression after discontinuing the medication [13]. The risk of this toxicity is cumulative and dependent on the daily dose of the medication and its duration of use. The risk of retinal toxicity in the first 5 years of use is low and generally under 1%. In the first 10 years this risk generally remains under 2%. But the risk increases over time, and in some studies is a high as 20% after 20 years of use [11]. As a result, rheumatologists, dermatologists, and ophthalmologists have traditionally worked together to manage patients on HCQ therapy and to mitigate these risks.

In 2016, the American Academy of Ophthalmology (AAO) issued updated guidelines for the dosing of HCQ and the ophthalmologic screening recommendations for these patients [14]. The research informing these guidelines included the above findings that the toxicity is not rare among long-term users of the medication, and that the risk for retinopathy is dependent on the daily dose in proportion to the patient’s actual body weight (ABW) (as opposed to ideal body weight [IBW]) [11]. These guidelines also argued that a lower risk was achieved with daily doses of HCQ < 5 mg/kg/day, based on ABW [11]. These ABW-based guidelines especially mitigated long term risk, reducing the risk at 10 and 20 years of exposure to < 2% and < 4% respectively [14].

However, despite these guidelines, research shows that rheumatologists and dermatologists continue to prescribe daily doses of HCQ that often exceed these recommendations [15]. Given this discrepancy, we wanted to assess HCQ prescribing habits and rates of adherence with these guidelines at our academic institution. We also wanted to use survey data to better understand prescribers’ opinions on these guidelines. Finally, as previous research has demonstrated improved rates of adherence with these dosing guidelines when auto-dosing functions are introduced into the electronic medical record (EMR), we conducted a prospective, non-controlled study to determine if a new CDS tool in the EMR, could affect adherence with these guidelines [16].

## Methods

### Study design and populations

Survey data and chart data was collected before (June 2017 – January 2019) and after (March 2019 – January 2020) our CDS intervention. Data was collected from the rheumatology and dermatology clinics of the University of Utah Medical Center in Salt Lake City, Utah, USA.

Chart data was taken from the EMR of outpatient clinic encounters. Through the University of Utah’s enterprise data warehouse, non-identified data was queried from all rheumatology and dermatology encounters which were documented during these two time periods and in which HCQ was prescribed as part of the visit. If a patient had more than one outpatient encounter that met these criteria, then the most recent encounter was used. Encounters were linked to the associated Department; HCQ prescriptions from dermatology encounters were managed by dermatology and HCQ prescriptions from rheumatology encounters were managed by rheumatology. Data queried included: the most recently recorded weight (even if this was not associated with the encounter in question), recorded sex, the diagnosis linked with the HCQ prescription in the EMR, and other available prescriptions details associated with the HCQ order. Other than sex, no patient identifying data was collected. If no weight was on record, or if other prescription details were missing that prevented us from being able to calculate the daily dose of HCQ, then the record was removed from further analysis. Data assimilation and organization was conducted (RO) for subsequent statistical analysis (RO, GS, JP).

Survey data was also collected before and after our CDS intervention. Potential features of the pre-intervention and post-intervention surveys were explored (RO, DO, CH, JP), the surveys were then written (RO) and reviewed before use (RO, DO, CH, JP). No pilot testing or cognitive interviewing was conducted. These surveys were not further validated. Pre-intervention surveys were anonymous paper surveys for rheumatologists (distributed and collected by an administrative assistant not associated with the study). Due to lack of proximity, the same. Anonymous surveys were instead e-mailed to dermatologists. Post-intervention surveys were anonymous e-mailed online surveys for both rheumatologists and dermatologists, due to ease of distribution and anonymity. Every member of our rheumatology and dermatology divisions (faculty, advanced practitioners, and fellows) were asked to participate in the surveys, both before and after our intervention. Participation was voluntary. The surveys consisted of multiple-choice or yes/no questions. Survey data analyzed included: self-reported awareness of guidelines, multiple choice questions regarding dosing recommendations of the guidelines, self-reported adherence with guidelines, and opinions regarding these guidelines (full surveys are available in the supplemental materials).

Our study received an institutional review board (IRB) exemption at the University of Utah (IRB 00132645). A consent cover letter was provided to providers for completion of anonymous surveys. All methods were carried out in accordance with relevant guidelines and regulations.

### Intervention

Our intervention was the implementation of a CDS tool. This tool was an alert in the EMR in any outpatient encounter when a provider ordered the medication HCQ. This alert would prompt the provider, when choosing the dose signature, to choose a calculated 5 mg/kg/day dose. This alert also simultaneously highlighted a reference to the 2016 AAO guidelines supporting this recommendation. If no weight was on record in the chart, an error message resulted. Alternative options included to choose 400 mg daily or to enter an alternative dosing schedule manually. If a weight was on record in the chart, and the prescribed value (regardless of the option used) exceeded 5 mg/kg/day, then the provider received a dose warning message. This tool was not a “hard stop”. Providers were not required to select the recommended weight-based dose and were not required to justify their reasons for prescribing a higher dose. The only recorded variable for analysis was the final daily dose. However, we were able to determine if the prescriber had used this CDS 5 mg/kg/day prompt to choose a dose or had instead opted to enter a dose manually.

### Measurement and outcomes

We analyzed HCQ prescribing habits of the providers and their adherence with the 2016 AAO guidelines. We also collected the providers’ opinions and reasons for practice deviations from the guidelines in survey form. Finally, we evaluated whether introduction of the CDS tool correlated with any change in the rate of adherence and providers’ perspectives on the guidelines.

### Statistical analysis

Demographic data and ABW-based HCQ dosing prescriptions were presented as frequencies and percentages. Comparisons of proportions before and after the intervention were calculated using Fisher’s exact tests, and comparisons of means were calculated using T-test. Stata 16 software was used. The *P*-value of < .05 was considered statistically significant.

We modeled the ordered categorical weight-based doses (≤ 5.0 mg/kg/day, 5.1–6.5 mg/kg/day, ≥ 6.6 mg/kg/day) using multivariable mixed effects ordinal logistic regression controlling for specialty (rheumatology versus dermatology), age, sex, weight, diagnosis (rheumatoid arthritis and lupus were compared as a group, compared to all other diagnoses), and provider. We included provider identification as a random effect to account for prescriptions clustered within provider. After dichotomizing the weight-based dose categories (goal dose versus ≥5.0 mg/kg/day) we fit a multivariable mixed effects Poisson regression model with a robust standard error controlling for these same variables. Stata 17 software was used. The *P*-value of < .05 was considered statistically significant.

## Results

### Pre-intervention analysis (June 2017–January 2019)

**Fig. 1:**
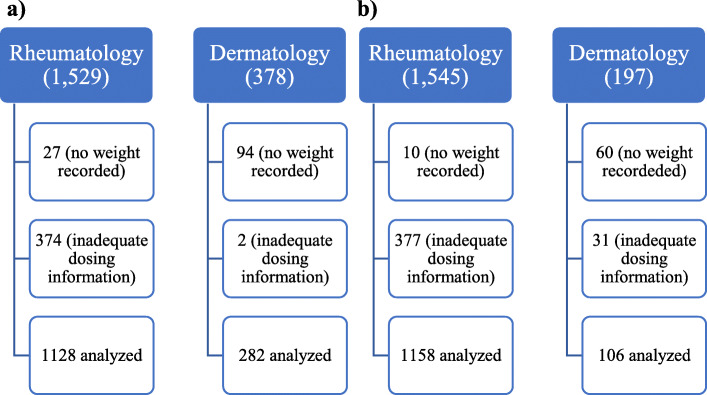
Pre-intervention (**a**) and Post-intervention (**b**) Chart Review

One thousand one hundred twenty-eight rheumatology encounters were available for analysis (Fig. 1). Of these, 778 (68.9%) were prescribed ≤5 mg/kg/day; 277 (24.6%) were prescribed 5.1–6.5 mg/kg/day; 73 (6.5%) were prescribed ≥6.6 mg/kg/day. In total 350 (31.0%) rheumatology patients were prescribed an HCQ dose above the recommended limit (Table 1).

**Table 1:** Hydroxychloroquine dosing pre-intervention versus post-intervention

HCQ Dose (mg/kg/day)	Pre-intervention		Post-intervention	
	Rheumatology, *n* (%) *n* = 1128	Dermatology, *n* (%) *n* = 282	Rheumatology, *n* (%) *n* = 1161	Dermatology, *n* (%) *n* = 110
≤ 5.0	778 (68.9)	170 (60.3)	894 (77.0)	82 (74.5)
Female	626 (80.555.5)	122 (71.843.3)	708 (79.261.0)	56 (68.350.9)
Lupus^a^	148 (23.613.1)	12 (9.8 (4.3)	79 (11.26.8)	9 (16.18.2)
RA	201 (32.117.8)	0	133 (18.811.5)	0
Male	152 (19.513.5)	48 (28.217.0)	186 (20.816.0)	26 (31.723.6)
Lupus^a^	16 (10.51.4)	10 (20.83.5)	11 (5.90.9)	6 (23.15.5)
RA	64 (42.15.7)	0	55 (29.64.7)	0
5.1–6.5	277 (24.6)	78 (27.7)	222 (19.1)	22 (20.0)
Female	256 (92.422.7)	65 (83.323.0)	197 (88.717.0)	19 (86.417.3)
Lupus^a^	70 (27.36.2)	10 (15.43.5)	20 (10.21.7)	3 (15.82.7)
RA	61 (23.85.4)	0	38 (19.33.3)	0
Male	21 ((1.9)7.6)	13 (16.74.6)	25 (11.32.2)	3 (13.62.7)
Lupus^a^	4 (19.00.35)	3 (23.11.1)	2 (0.10.2)	1 (33.30.9)
RA	11 (52.40.98)	0	10 (0.40.9)	0
≥ 6.6	73 (6.5)	34 (12.1)	45 (3.9)	6 (5.5)
Female	70 (95.96.2)	32 (94.111.3)	42 (93.33.6)	4 (66.73.6)
Lupus^a^	17 (24.31.5)	3 (9.41.1)	2 (4.80.2)	0
RA	17 (24.31.5)	0	5 (11.90.4)	0
Male	3 (4.10.27)	2 (5.90.71)	3 (6.70.3)	2 (33.31.8)
Lupus^a^	0	0	0	0
RA	2 (66.70.18)	0	2 (66.70.2)	0

*RA* rheumatoid arthritis

^a^ Includes systemic lupus erythematosus, discoid lupus, acute cutaneous lupus, glomerular disease associated with lupus

Two hundred eighty-two dermatology encounters were available for analysis (Fig. 1). Of these, 170 (60.3%) were prescribed ≤5 mg/kg/day; 78 (27.7%) were prescribed 5.1–6.5 mg/kg/day; 34 (12.1%) were prescribed ≥6.6 mg/kg/day. In total 112 (39.7%) dermatology patients were prescribed an HCQ dose above the recommended limit (Table 1).

Combining rheumatology and dermatology patients, 462 (32.8%) were prescribed an HCQ dose above the recommended limit. There were significantly fewer number of patients in rheumatology encounters compared to dermatology encounters prescribed an HCQ dose above the recommended limit (*P* = .007).

In rheumatology patients who received an HCQ dose ≥6.6 mg/kg/day, the indication was most often for RA (*n* = 19) and SLE (*n* = 17). In dermatology patients, the indications for this higher dose included lichen planus (*n* = 4), discoid lupus (*n* = 2), dermatomyositis (*n* = 1), granuloma annulare (*n* = 1), and urticaria (*n* = 5).

We compiled 18 surveys from rheumatology and 12 surveys from dermatology (30 surveys total). Most participants agreed with the guidelines, however a large portion also expressed concern that adjusting the HCQ dose could lead to non-adherence or a flare of disease. As much as 10% of providers admitted to not using these guidelines in their clinical decision making, and 15% chose incorrect answers suggesting they did not know the recommendations in these guidelines (Table 2).

**Table 2:** Survey Responses on Hydroxychloroquine Dosage and Guidelines (Pre-intervention)

Reasons	Rheumatologists, *n* (%) (total = 18)	Dermatologists, *n* (%) (total = 12)	Total, *n* (%) (total = 30)
Chose incorrect weight-based dose	3 (16.7)	3 (25.0)	6 (20.0)
Chose ideal body weight rather than actual weight	5 (27.8)	4 (33.3)	9 (30.0)
Report not using the guidelines	2 (11.1)	1 (8.3)	3 (10.0)
Express concern for non-adherence if changing the dose	6 (33.3)	5 (41.7)	11 (36.7)
Express concern for disease flare if changing the dose	10 (55.6)	4 (33.3)	14 (46.7)
Believe an EMR tool would change their practice	9 (50.0)	8 (66.7)	17 (56.7)
Agree with the guidelines	13 (72.2)	11 (91.7)	24 (80.0)

### Post-intervention analysis (march 2019–April 2020)

One thousand one hundred sixty-one rheumatology encounters were available for analysis (Fig. 1). Of these, 894 (77.0%) were prescribed ≤5 mg/kg/day; 222 (19.1%) were prescribed 5.1–6.5 mg/kg/day; 45 (3.9%) were prescribed ≥6.6 mg/kg/day. In total 267 (23.0%) rheumatology patients were prescribed an HCQ dose above the recommended limit (Table 1). 57 (4.9%) rheumatology patient encounters used 5 mg/kg/day doses prompted by the CDS tool.

One hundred ten dermatology encounters were available for analysis (Fig. 1). Of these, 82 (74.5%) were prescribed ≤5 mg/kg/day; 22 (20.0%) were prescribed 5.1–6.5 mg/kg/day; 6 (5.5%) were prescribed ≥6.6 mg/kg/day. In total 28 (25.5%) dermatology patients were prescribed an HCQ dose above the recommended limit (Table 1). 3 (2.7%) dermatology patient encounters used 5 mg/kg/day doses prompted by the CDS tool. Combining rheumatology and dermatology patients, 295 (23.2%) were prescribed an HCQ dose above the recommended limit. There was no difference in the number of patients in rheumatology encounters compared to dermatology encounters prescribed an HCQ dose above the recommended limit (*P* = .56).

In rheumatology patients who received an HCQ dose ≥6.6 mg/kg/day, the clinical indication was most often for RA (*n* = 7) and Sjogren’s syndrome (*n* = 4). In dermatology patients, indications for this higher dose included unspecified dermatitis (*n* = 1), lichen planus (*n* = 1), and chilblain lupus (*n* = 1).

We compiled 16 surveys from rheumatology and 12 surveys from dermatology (28 total). Most providers agreed with the guidelines. 25 and 46.4% respectively reported that the CDS tool changed their practice and was helpful. However, 35.7% reported being unaware of the CDS tool entirely. 17.9% report not using the guidelines, and 42.8% still chose incorrect answers suggesting they did not know the recommendations in these guidelines (Table 3).

**Table 3:** Survey Responses on Hydroxychloroquine Dosage and Guidelines (Post-intervention)

Reasons	Rheumatologists, *n* (%) (total = 16)	Dermatologists, *n* (%) (total = 12)	Total, *n* (%) (total = 28)
Chose incorrect weight-based dose	0 (0)	3 (25.0)	3 (10.7)
Chose ideal body weight rather than actual weight	4 (25.0)	5 (41.7)	9 (32.1)
Report not using the guidelines	3 (18.8)	2 (16.7)	5 (17.9)
Express concern for non-adherence if changing the dose	3 (18.8)	2 (16.7)	5 (17.9)
Express concern for disease flare if changing the dose	7 (43.8)	4 (33.3)	11 (39.3)
Report being unaware of the CDS tool	5 (31.3)	5 (41.7)	10 (35.7)
Agree with the guidelines	11 (68.8	10 (83.3)	21 (75.0)
Report the CDS tool changed their practice	4 (25.0)	3 (25.0)	7 (25.0)
Report the CDS tool is helpful/beneficial	8 (50.0)	3 (25.0)	13 (46.4)

### Pre-intervention versus post-intervention analysis

There was no difference in sex between the pre-intervention group versus the post-intervention group; neither for the entire cohort (*P* = .12), the rheumatology cohort (*P* = .08), nor the dermatology cohort (*P* = .24). In the entire cohort, there was a significant difference in weight between the pre-intervention group (*M* = 80.0 kg, *SD* = 22.7) and the post-intervention group (*M* = 83.1 kg, *SD* = 21.9) (*P* < .001). In the rheumatology cohort, there was a significant difference in weight between the pre-intervention group (*M* = 77.1 kg, *SD* = 22.4) and the post-intervention group (*M* = 79.4 kg, *SD* = 21.5) (*P* = .01). In the dermatology cohort, there was a significant difference in weight between the pre-intervention group (*M* = 74.8 kg, *SD* = 23.8) and the post-intervention group (*M* = 86.1 kg, *SD* = 20.4 (*P* < .001). There were significantly more patients with a diagnosis of lupus (SLE, discoid lupus, other cutaneous lupus, or glomerular or organ disease from lupus) in the pre-intervention group (20.8%) compared to the post-intervention group (10.5%) (*P* < .001). There were significantly more patients with a diagnosis of rheumatoid arthritis in the pre-intervention group (25.2%) compared to the post-intervention group (19.1%) (*P* < .001).

For rheumatologists, 350/1128 (31.0%) prescribed an HCQ dose > 5 mg/kg/day in the pre-intervention analysis and 267/1161 (23.0%) did so in the post-intervention analysis; a statistically significant decrease of 8.0% (*p* = 0). For dermatologists, 112/282 (39.7%) prescribed an HCQ dose > 5 mg/kg/day in the pre-intervention analysis and 28/110 (25.5%) did so in the post-intervention analysis; a statistically significant decrease of 14.2% (*p* = .01). When rheumatologists and dermatologists are combined, 462/1410 (32.8%) prescribed an HCQ dose of > 5 mg/kg/day in the pre-intervention analysis and 295/1271 (23.2%) did so in the post-intervention analysis; a statistically significant decrease of 9.6% (*P* < .001).

Using multivariable mixed effects ordinal logistic regression model, the odds of a patient receiving a higher dose of hydroxychloroquine in the post-intervention period, relative to the pre-intervention period, was 0.69 (OR = 0.69, 95% CI: 0.57–0.83, *P* < .001) after controlling for specialty (rheumatology versus dermatology), age, sex, weight, diagnosis (rheumatoid arthritis and lupus versus neither), and provider. When controlling for the associated provider, we found no additional effect on dosing habits from pre-intervention to post-intervention dosing. Using multivariable mixed effects Poisson regression with a robust standard error, the adjusted percent of prescriptions of hydroxychloroquine that were ≤ 5.0 mg/kd/day was 75.5% in the post-intervention period compared to 68.2% in the pre-intervention period (RR 1.11, 95% CI 1.06–1.16, *P* < .001), after adjusting for these same variables [17].

**Fig. 2:**
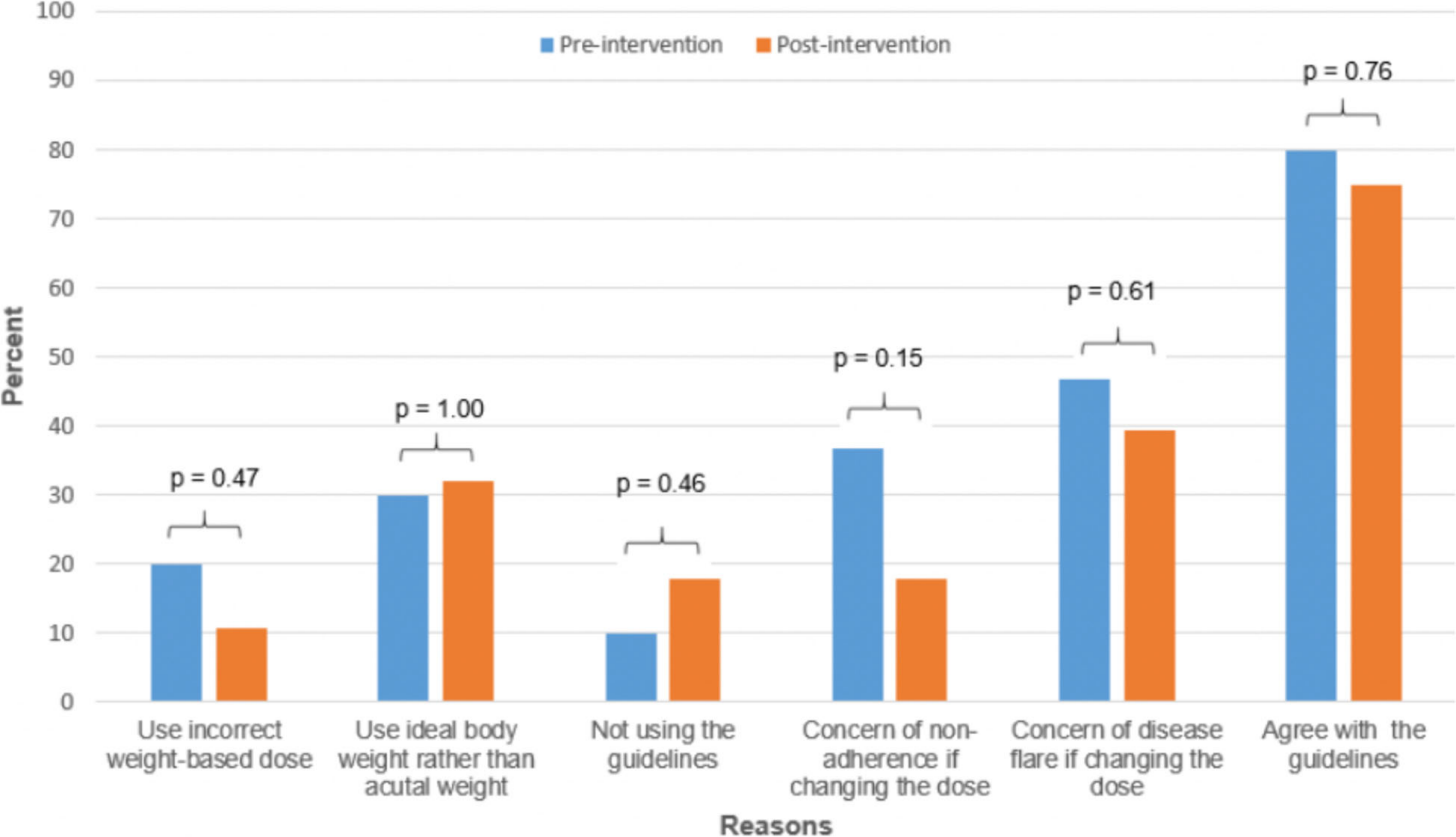
Reasons for Incorrect Hydroxychloroquine Dosage and Opinions on the Guidelines Before and After the Intervention

There were reductions in the number of rheumatologists who incorrectly identified the guideline-based weight-based dosing recommendation of HCQ after our intervention; there was no change in dermatologists. When these two groups are combined and analyzed as a whole, there was an overall reduction but not of statistical significance (*P* = .47). After our intervention, there were reductions in the reported concern for non-adherence, and the reported concern for a disease flare, which did not reach statistical significance. Otherwise, there were no differences in the additional surveyed questions exploring reasons for choosing an HCQ dose and opinions on these guidelines; not when comparing before and after the intervention nor between rheumatology and dermatology (Fig. 2).

## Discussion

This study reveals that there is an enduring gap between providers’ intended practice and actual practice in weight-based dosing of HCQ at our academic institution. The majority of surveyed providers profess using the guidelines in their clinic practice while prescribed doses often exceed the recommended daily dose (a frequency as high as 32.8% in this study.) This could be attributed to a misunderstanding or unfamiliarity with the 2016 guidelines, as 10.7–32.1% of survey respondents could not correctly identify the specifics of these dosing guidelines. A similar lack of assimilation or understanding of other aspects of these guidelines has been demonstrated elsewhere [18]. There may also be more mundane pitfalls attributable to the logistics of a busy and rushed clinical practice, not measured here, which challenge good intentions. However, survey data also suggested that this discordance may be due to providers’ concern that changes in the prescribed dose of HCQ could lead to disease flares (39.3–46.7%) or may contribute to non-adherence in their patients (17.9–36.7%). Additionally, there was concern that these guidelines do not adequately balance the risk of retinal toxicity with the therapeutic benefit of HCQ (20.0–25.0%).

The fact that females were more often prescribed a higher and potentially toxic weight-based dose has been documented elsewhere and is presumably a multi-factorial phenomenon [19]. First, females are more likely to weigh less than their male counterparts. Thus females, like children and the elderly, are at risk for over-dose or toxicity when population standard dosing protocols are applied (such as the previous guidelines recommending HCQ at 400 mg daily for most patients). Alternatively, patients on higher doses may be suffering more severe disease, (although there is no way to verify this in our data) and more severe disease may persuade a provider to prescribe higher doses of HCQ, even if only temporarily, as the acute risk of the autoimmune disease outweighs the long-term risk of retinal toxicity.

An unexpected finding in this research was the number of patients who were prescribed HCQ without a contemporaneously or previously recorded weight. This suggests that even when most providers are familiar with the clinical guidelines, the lack of a recorded weight, perhaps as a systems failure in our clinical structure, is a variable which could prohibit guideline adherence in actual clinical practice. This was especially noted in the dermatology encounters where 30% of encounters were with patients for whom no weight was recorded in the EMR. Based on the findings of this research, re-designed workflow or end-user education might improve the availability of this data to guide appropriate dosing recommendations.

In formulating our CDS tool, we attempted to target an intervention with a high likelihood of success. Emphasis was placed on a simple intervention that would fit seamlessly into the providers’ normal workflow. Our tool did not present an additional “step” or “pop-up,” but was instead integrated into the EMR flow of a HCQ prescription. Interaction with this tool was fast with no expectation that it would slow or hinder clinic work for providers [20]. As such, most providers surveyed believed that the CDS tool was useful (72.2%). This proves that a CDS intervention of this nature is accepted and tolerated by providers. Previous research has suggested that CDS interventions can influence provider behaviors regarding guideline adherence [21]. We show here that our CDS intervention was associated with a decrease in the percentage of patients prescribed HCQ doses that exceed dosing guidelines, both in the rheumatology and dermatology clinical context. This further supports that an intervention such as our CDS tool can improve adherence with these guidelines and thus improve the long-term safety of our patients.

A topic not addressed by this research is how the recommendations and weight-based dosing may affect drug level measurements in patients and how this may relate to drug efficacy and the long-term risk for toxicity. This is an important component of the ongoing debate regarding best dosing practices of HCQ, especially in the SLE population [22].

There are weaknesses in our study. First, we have a limited sample size of providers which limits our ability to identify differences in the rate of concordance with the 2016 guidelines. Additionally, there are weaknesses inherent to our survey data. Specifically, providers were only able to confirm or deny the investigators suggested reasons for possible non-adherence with these guidelines (surveys were multiple choice). Qualitative research would be an interesting next step towards more thoroughly understanding any hesitancies or barriers to adherence with these guidelines. We do not know the sensitivity and specificity of our surveys and they were not rigorously validated. Additionally, we observed changes in prescribing habits, which correlated with our CDS tool intervention; however, a causative relationship cannot be concluded from this research. The very nature of our pre-intervention survey could have increased recognition and understanding of these guidelines, rather than our CDS intervention. It is also possible that adherence to guidelines takes time and follows a “natural history” of guideline acceptance that might be reflected in this improvement over the course of one year. However, evidence shows us that active interventions are required to enhance guideline uptake and our results seem to provide another example of this finding [23, 24]. Importantly, this is not a longitudinal study but is a series cross-sectional analysis. This again limits our ability to understand any possible or suggested causality. Finally, there are logistical reasons having to do with data collection from the EMR itself. Our prescribing practices were those as defined by the prescription history. However, prescribers might communicate directly with their patients to outline a different dosing schedule or to change the dosing schedule over time. Additionally, some indications may warrant a higher dose of HCQ at diagnosis, followed by a taper after control of disease; in a cross-sectional analysis, this data would not be captured. This importantly would alter our interpretation of the data, and more importantly it is not well understood what if any increased risk for retinal toxicity this practice might incur a particular patient.

## Conclusions

We believe that this research provides several important take home points and contributes significantly to our understanding of current HCQ prescribing practices and opinions on the associated AAO guidelines. First, this research highlights ongoing hesitation of some rheumatologists and dermatologists, in an academic medical center, towards adherence with the 2016 AAO recommendations for HCQ ABW-based dosing. Second, this research demonstrates ongoing unfamiliarity with the details of the recommendations therein and discrepancy between the perceived and the actual knowledge of prescribers. Despite prescribers’ declared familiarity with the 2016 recommendations regarding weight-based dosing of HCQ, survey results suggest that not all providers are familiar with the specifics of the guidelines. Prescribed HCQ doses often exceeded the recommended daily dose. Finally, our research shows that a CDS tool, prompting safe and ABW-based dosing of HCQ, is well tolerated by providers and can improve adherence with these guidelines. We believe that this improvement will lead to greater patient safety over the lifetime.

Further research should be a combined effort of rheumatology, dermatology, and ophthalmology to explore tools, including CDS interventions, for bridging this gap between perceived guideline use and clinical implementation. Additional interesting extensions of this research should correlate the relationship with HCQ dosing guideline adherence and the incidence of disease flares and retinal toxicity in this patient population.

## Supplementary Information

**Additional file 1.** Post-intervention survey.

**Additional file 2.** Pre-intervention survey.

## Data Availability

The datasets used and/or analyzed during the current study are available from the corresponding author on reasonable request.

## Abbreviations

AAO: American academy of ophthalmology
CDS: Clinical decision support
DMARD: Disease modifying anti-rheumatic drug
EMR: Electronic medical record
HCQ: Hydroxychloroquine
IRB: Institutional review board
QI: Quality improvement
RA: Rheumatoid arthritis
SLE: Systemic lupus erythematosus
TLRs: Toll-like receptors
