# Supplementary material for: The effect of an electronic medical record intervention on hydroxychloroquine prescribing habits and surveyed providers’ opinions of the 2016 American Academy of Ophthalmology guidelines in the rheumatology and dermatology practices of an academic institutionle
Source: BMC Health Serv Res. 2021 Sep 3;21:913. doi: 10.1186/s12913-021-06954-8 (PMC8418105; doi:10.1186/s12913-021-06954-8)
Supplement: Supplementary file 1 — Additional file 1. Post-intervention survey. [file 12913_2021_6954_MOESM1_ESM.docx]

Post-intervention survey

1. What are the most recent recommendations for daily hydroxychloroquine dosing? *(Marmor, M.F., et al., Recommendations on Screening for Chloroquine and Hydroxychloroquine Retinopathy (2016 Revision). Ophthalmology, 2016. 123(6): p. 1386-94.)*
   1. ≤ 5.0mg/kg
   2. 5-6 mg/kg
   3. ≤ 6.5 mg/kg
2. Should the calculation in question #1 be based on actual body weight or ideal body weight?
   1. Actual
   2. Ideal
3. Do you use these 2016 guidelines when prescribing hydroxychloroquine to your patients?
   1. Yes
   2. No
4. Do you feel that these 2016 guidelines accurately balance the risk of retinal toxicity with the therapeutic benefit of hydroxychloroquine?
   1. Yes
   2. No
5. Are you concerned that changing your patients’ dose based on these guidelines might lead to non-adherence in your patients?
   1. Yes
   2. No
6. Are you concerned that changing your patients’ dose based on these guidelines might lead to flares of their auto-immune disease?
   1. Yes
   2. No
7. Are you aware that since March of 2019 the electronic medical record (Epic) has been automatically calculating a recommended weight-based dose, based on these 2016 guidelines, when prescribing or refilling hydroxychloroquine?
   1. Yes
   2. No
8. If you answered “yes” to question #7, do you find this automatic calculation feature helpful/beneficial?
   1. Yes
   2. No
   3. Not applicable
9. If you answered “yes” to question #7, did this automatic calculation feature influence or change your prescribing practice?
   1. Yes
   2. No
   3. Not applicable
10. Are you a member of the rheumatology team or dermatology team?
    1. rheumatology
    2. dermatology
